# A Transferable Digital Twin-Driven Process Design Framework for High-Performance Multi-Jet Polishing

**DOI:** 10.3390/mi17020226

**Published:** 2026-02-10

**Authors:** Honglei Mo, Xie Chen, Lingxi Guo, Zili Zhang, Xiao Chen, Jianning Chu, Ruoxin Wang

**Affiliations:** 1 Shanghai Aerospace Control Technology Institute, Shanghai 201109, China; mohonglei1987@sina.com (H.M.); spwanx@163.com (X.C.); guolingxiz@yeah.net (L.G.); 2 State Key Laboratory of Ultra-Precision Machining Technology, Department of Industrial and Systems Engineering, The Hong Kong Polytechnic University, Hong Kong 999077, China; zili1.zhang@polyu.edu.hk; 3 Hubei Key Laboratory of Modern Manufacturing Quality Engineering, School of Mechanical Engineering, Hubei University of Technology, Wuhan 430068, China; chenxiao1987jz@163.com (X.C.); jianning_chu@163.com (J.C.); 4 Department of Mechanical Engineering, University of Maryland College Park, College Park, MD 20742, USA

**Keywords:** digital twin, surface roughness prediction, fluid jet polishing, transfer learning, machining process

## Abstract

The multi-jet polishing process (MJP) demonstrates high shape accuracy and surface quality in the machining of nonlinear and complex surfaces, and it achieves precise and adjustable material removal rates through computer control. However, there are still challenges in terms of machining efficiency, system complexity, and stability. In particular, maintaining the polishing quality presents a greater challenge when working conditions change. To overcome these issues, this paper conceptually proposes a digital twin (DT)-driven, human-centric design framework that integrates key factors of MJP, such as jet kinetic energy, nozzle structure, abrasive type, and machining path. Within this framework, a feature-encoded transfer learning-based model is introduced to enhance surface roughness prediction accuracy and robustness under varying working conditions. The effectiveness of the proposed model was verified by conducting experiments on 3D printed workpieces under two different MJP working conditions. The results show that our proposed method yields better predictive performance and cross-condition adaptability. Overall, this work provides a predictive modeling component that supports DT-driven process design, offering a practical and extensible perspective for optimizing complex ultra-precision manufacturing processes under data-scarce and uncertainty-dominated conditions.

## 1. Introduction

Fluid jet polishing (FJP) is an ultra-precision manufacturing process that involves mixing water with abrasive particles and ejecting this mixture through a small nozzle at high velocities, thereby achieving high surface quality. Different to the contact machining methods such as diamond cutting [1,2] and grinding [3], the non-contact finishing technique is favored for its ability to achieve high-precision polishing without causing overheating of the workpiece. In addition, FJP also avoids the issue of tool wear and is capable of adapting to the processing requirements of complex nonlinear surfaces. With computer control, FJP can achieve accurate processing effects and controllable material removal rates, making it suitable for processing hard alloys, ceramics, glass, quartz, and other special materials. Due to its high-precision machining capabilities, FJP can handle complex freeform surfaces, thus becoming a dominant technology in the field of ultra-precision machining.

Despite the great potential of FJP technology in the field of precision machining, it still faces challenges and limitations, such as the need for improved processing efficiency, system complexity, and stability requirements. Moreover, most FJP systems use open-loop control systems that cannot effectively provide feedback during the polishing process to adapt to uncertainties during machining. Recently, with the development of intelligent manufacturing, many studies are emerging to address these issues [4,5]. Data-driven approaches have shown significant promise in predicting complex machining outcomes where physics-based models struggle. For instance, the recent precision manufacturing domain demonstrated the generalizing ability of convolutional neural network models in predicting workpiece profiles in electrochemical machining under varying pulse conditions [6]. Furthermore, recent advancements have focused on overcoming the “black-box” nature of these models. Wu et al. [7] introduced Explainable AI (XAI) frameworks to interpret model decisions in real-time cavity profile prediction, bridging the gap between data-driven predictions and physical process dynamics. In particular, digital twin (DT) technology has been widely applied in various fields, including product lifecycle management, processing, assembly processes, and predictive and health management. In the FJP process, DT technology has the potential to enhance the accuracy and efficiency of process simulation, optimization, and control by integrating real-time sensor feedback into simulation models and data analysis processes. Therefore, in-depth research into the application of DT technology in FJP, especially in terms of process parameters, material removal function models, and processing path algorithms, will be key to improving machining precision and efficiency.

In this paper, a DT-based method is proposed to optimize the multi-jet process (MJP), a more efficient FJP process. Firstly, recent research on DT in the field of machining is reviewed to identify the shortcomings of existing methods. The key factors of MJP, such as jet kinetic energy, nozzle design, abrasive type, processing path, and removal rate model, are analyzed. Subsequently, a concept of a DT-based human-centric interaction design framework for the MJP process is proposed. In this framework, a feature encoding-based transfer learning method is designed to realize efficient, precise, and stable polishing process quality prediction under various working conditions. The effectiveness of the proposed method is validated through polishing experiments on the 3D printed material.

## 2. Related Works

### 2.1. Digital Twin in Machining

As the manufacturing industry transforms towards intelligent manufacturing models, DT-driven processing systems stand out for their capabilities in sensing, predicting, decision-making, and controlling. These systems have three characteristics: (1) the integration of various DT models, (2) the systematic operations that are constituted by interactions between these models, and (3) the provisioning of diverse services through the combination of these operational processes. The analysis of DT-driven machining can be approached from three perspectives: component (point), connectivity (line), and loop (face).

**(a) Component Perspective:** DT models serve as high-fidelity virtual counterparts to physical entities. They are influenced by differing manufacturing and process requirements and typically include geometric models, knowledge models, and algorithmic models [8]. Geometric models represent the changing geometric states and physical alterations of an entity [9]. Knowledge models uncover complex data correlations within the machining process and support various functionalities like algorithm development and process optimization [10]. Algorithmic models are crucial for forecasting the actual production process and enabling dynamic and real-time modifications to the machining process [11].

**(b) Connectivity Perspective:** Examining the flow and transformation of data unveils that DT machining systems embody three critical operations: from physical to digital (sensing), virtual analysis (cognition), and from digital back to physical (feedback and interaction). Sensing involves the real-time acquisition of physical data to support decision-making [12]; cognition is the process of acquiring knowledge or predictive information [13]; feedback generates or displays actions that alter the physical world [14]; and interaction encompasses human participation within a looped environment [15].

**(c) Loop Perspective:** The operation of a DT machining system is cyclical, providing looped services tailored to various manufacturing stages, including process planning, autonomous decision control, human-in-the-loop control, and quality inspection. Process planning integrates various factors and verifies simulation results through actual machining to improve quality [16]. Autonomous decision control adjusts and fine-tunes the machining process based on the sensed information [17]. Human-in-the-loop control refers to intelligent decision-making by on-site personnel with the assistance of human–machine interactions [18]. DT-driven quality inspection involves analyzing production data to monitor product quality [19].

In summary, DT technology, with its comprehensive models, operations, and services, offers an integrated and dynamically optimized environment for intelligent manufacturing, enhancing processing efficiency, quality, and flexibility. This advancement is unlocking transformative potential in the manufacturing sector. A focus on the loop perspective, particularly the process design phase and related decision models within the DT machining system, will be crucial in future explorations.

### 2.2. Digital Twin-Driven Process Design

The main aim of intelligent manufacturing is to enhance manufacturing performance. Consequently, post-designing a product that conforms to aesthetic standards, it is crucial to conduct manufacturing simulations to ascertain if the product’s performance aligns with the anticipated benchmarks. Furthermore, it is imperative to evaluate the machining process to establish whether any modifications are necessary. Therefore, process design is crucial and important. The scope of research in process design spans products, processes, and systems. Utilizing iterative design outcomes during the process design phase can bolster the quality of machining. Product design is largely concerned with devising product functionality and the allocation of product tolerances [20]. In addition, process design is inherently intricate, demanding a holistic assessment of diverse elements, including the workpiece, machining equipment, and parameters [21].

For the design of the manufacturing process, DT plays a pivotal role in offering services such as mapping out manufacturing routes [22], optimizing parameters [23], designing eco-friendly processes [24], reapplying knowledge [25], and evaluating procedures [26]. In system analysis, researchers have delved into methods for appraising spindle performance, strategies for optimizing processes in response to fluctuating operational conditions and assessing the system’s compatibility during the design phase [27]. These design instances, facilitated by iterative features, have demonstrated that DT-led design paradigms can significantly enhance the performance of products, processes, and systems.

### 2.3. Research Gaps and Motivations

Although many studies have explored DT-driven machining processes, several research gaps remain in the context of ultra-precision machining:

(1) While DT technology has been recognized as an important technique to enhance the intelligence of manufacturing systems, design-level DT-driven frameworks for ultra-precision machining remain underexplored.

(2) Many studies on the method of machining process design already exist [28], but most of these methods are severely constrained by the original data collection scenarios. Therefore, their predictive performance and applicability often degrade significantly when process conditions change. Currently, there is a lack of effective modeling strategies, making it impossible to reuse previous knowledge and adapt to new processes or working conditions.

Therefore, in this paper, we propose a novel human-centric process design framework and a cross-domain adaptation mechanism to fill the research gaps mentioned above.

## 3. Digital Twin-Driven Process Design Method for Multi-Jet Polishing

### 3.1. Multi-Jet Polishing Process

**Figure 1:**
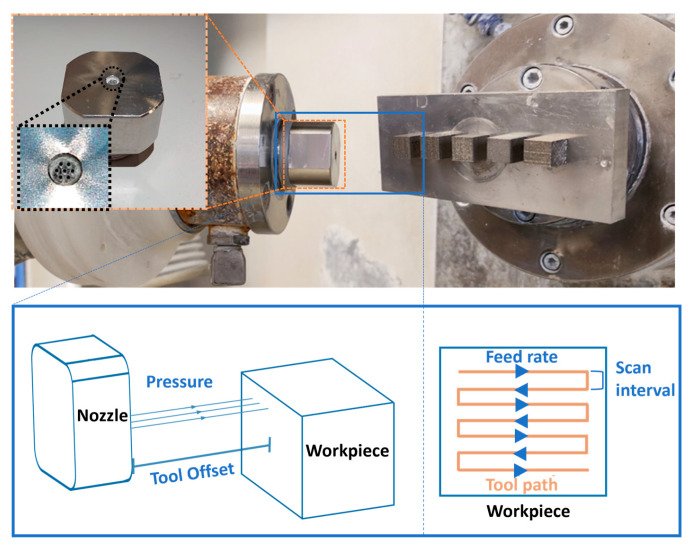
Mechanism of MJP.

MJP is a type of FJP, which utilizes multiple nozzles to improve polishing efficiency and uniformity. The fundamental mechanism of MJP is illustrated in Figure 1. It adopts multiple pressurized polishing slurry jets to improve the surface finishing where abrasive particles are accelerated to impinge on the workpiece surface, leading to nano-scale material removal. Basically, the surface generation of MJP is determined by both the Tool Influence Function (TIF) and the tool path planning. The TIF is assessed in terms of maximum depth, width and volumetric material removal rate, and can be affected by various polishing parameters including the abrasive particle concentration, slurry pressure, nozzle geometries, tool offset distance, impingement angle, etc. Two main factors that affected the impinging velocity and impact force of abrasive particles on the target surface were examined which are the fluid pressure (P) and tool offset distance (TO). Theoretically, higher pressure and smaller tool offset distance result in deeper material removal. Despite the TIF, tool path planning is also a key factor to the surface generation in MJP. The surface roughness profile of the polished surface is formed by the continuous repetitive impact of abrasive particles along the raster path under a specific tool feed rate (f) and step distance (d). The feed rate had controlled the dwelling time for material removal while the step distance controlled the overlapping area of the removal profiles. The detailed illustration is shown in Figure 1. The parameters of MJP can be outlined as follows:

**Kinetic Energy Parameters:** The kinetic energy of the jet is principally manifested through the energy and velocity of the abrasive particles. The greater the energy and velocity imparted to these particles are, the more significant their effect on the workpiece’s surface is. To augment the efficiency of material removal and the precision of the machining process, a thorough investigation is imperative for the sensitive parameters that influence the kinetic energy of the abrasives, such as the jet’s pressure, angle, standoff distance, and abrasive characteristics.

**Nozzle Design:** The design, quantity, and motion pattern of the nozzles in MJP play a substantial role in the machining performance. A qualitative analysis of the nozzle performance is necessary for achieving high surface quality.

**Abrasives:** The factor of the abrasives, such as shape, size, and type, have a direct impact on the efficiency of the machining process and the surface quality.

### 3.2. The Framework for Digital Twin-Driven Process Design Method

**Figure 2:**
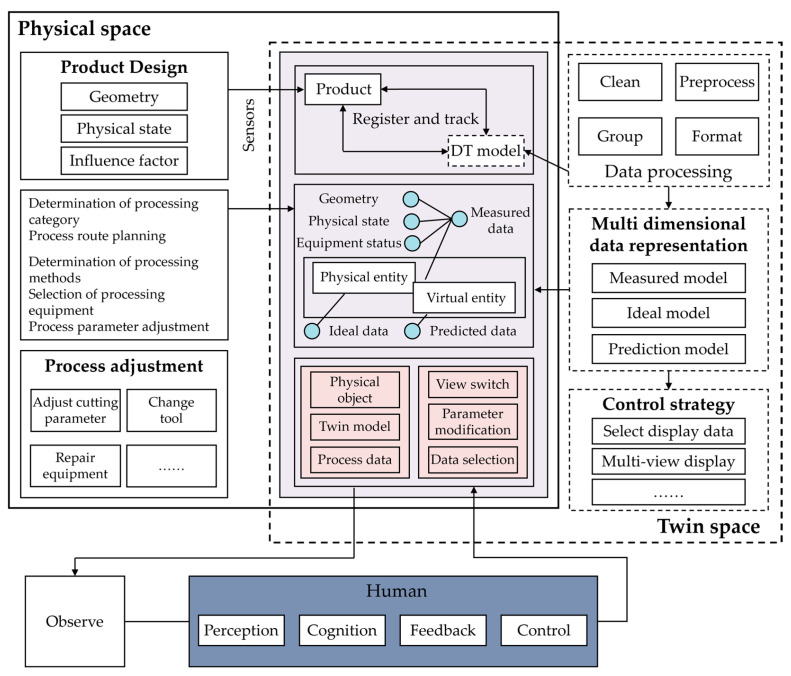
Conceptual architecture of a human-centric, DT-driven process design framework. The DT is to operate at the process-design level, where experimental data update the virtual model and predicted quality metrics provide feedback for process parameter optimization in a human-in-the-loop manner.

The dynamic nature of machining environments, where processes evolve over time, often leads to a decrease in the efficiency of process planning execution. Before implementing a process, it is crucial to evaluate whether the current process content meets the machining requirements of the workpiece and can produce a finished product of specified quality. Furthermore, it is essential to perform optimization calculations on real-time data and critical parameters during the process implementation, to provide a preselection of the optimal parameters and effective numerical references. Therefore, a human-centric, DT-driven process design framework is conceptually introduced, as illustrated in Figure 2. In the proposed framework, process data collected from physical polishing experiments, including process parameters and measured surface roughness, are used to update a virtual predictive model at the process-design level. Based on the updated model, predicted quality metrics and candidate parameter suggestions can be generated to support subsequent process planning and decision-making. Human designers remain in the loop and interpret these predictions when selecting or adjusting process parameters. In the proposed human-centric design approach, the DT model serves as the sole data source during the three-dimensional process design, playing a vital role in conveying process information. The core of the DT-driven human-centric process design method is the generation of an interactive process model based on the design model. The process design enables the creation of a three-dimensional solid process model that can manage process information. The automatic extraction of design model information provides a foundational layer of information. The construction of a process knowledge base, consisting of process instance libraries, process rule libraries, and equipment resource libraries, supports the reuse of process instances and automatic process decision-making within the aid of process design systems. Automatic process decision-making includes selecting machining methods, equipment, planning process routes, computer numerical control (CNC) machining operations, and computing decisions to achieve knowledge-based automatic planning of process routes and programmatic calculation of process parameters, generating comprehensive and detailed process information. Finally, the generated process information is integrated into a process information tree. Combined with model lightweight processing, a three-dimensional operation manual is created to realize visual guidance on the shop floor.

Based on the DT-driven process design approach in Figure 2, experiments are conducted in an iterative manner. First, an initial set of feasible process parameters is selected based on machine constraints and expert experience. Second, experiments are conducted on the physical system under the selected settings. Third, the surface roughness is measured after polishing and used as quality feedback. Fourth, the collected process quality data are incorporated to update the DT-driven prediction model. Finally, the updated model provides quality predictions under candidate parameter settings, which supports selecting the next trial settings for subsequent experiments.

Therefore, compared to traditional process evaluation methods, the human-centric process design approach has the following characteristics:

**Multi-Modal Process Information Perception:** The perception of the machining process relies on the equipment configured on-site to collect data. However, the fidelity of this perception depends heavily on understanding the process context (e.g., sensor positioning and signal source motion). As demonstrated by Wu et al. [29] in laser powder bed fusion, acoustic signals can vary significantly based on the relative position of the emission source, necessitating data-driven methods to identify these contextual factors for accurate monitoring. Consequently, the resulting massive twin dataset consists of multi-source heterogeneous data mappings that must be contextually aligned before being transmitted to the digital twin, enabling the virtual model to accurately reflect the physical process state. These data are used to continuously update the virtual process model, enabling synchronization between physical experiments and the DT at the process design level. The human-centric process design model supports these twin data, achieving optimization through machining quality prediction and refining machining paths, process methods, and process parameters.

**Quality-Centric Approach:** The entire process is quality-driven, with continuous adjustments made based on quality metrics derived from the virtual simulations and real-time data analysis, ensuring that the final product meets the specified standards. In this manner, predicted quality indicators serve as feedback from the DT to guide process design.

**Dynamic Adaptation:** The combination of real-time data with simulation allows for dynamic adaptation of the process, ensuring that any deviations from the desired outcomes can be corrected promptly, leading to a more agile and resilient manufacturing process. This adaptability is further enhanced by transferring knowledge learned from previous working conditions.

**Proactive Problem-Solving:** By placing humans in the loop, the design method allows for preemptive actions to be taken, ensuring that any potential issues can be anticipated and mitigated effectively, thus reducing downtime and enhancing the overall efficiency of the process.

**Human-In-The-Loop Design Mode:** The system uses virtual simulation technology to explore and predict the unknown world, followed by process modifications based on quality information until process execution is completed. Process designers can take proactive measures to avoid or solve potential disturbances in advance. This forms a closed decision-making loop between the physical process, the DT, and human designers.

### 3.3. The Adaptability of Process Design

In manufacturing industry, the adaptability of process design is crucial for ensuring product performance and manufacturing efficiency. Current research indicates that due to the complexity of manufacturing environments and the variability of process parameters, static process design models struggle to meet the rapidly changing market demands. Fluctuations in the environment, changes in material properties, and differences in operator skills are key factors that affect the stability of process design and product performance. Moreover, to meet the demands for personalized and customized products, process design must be flexible to accommodate continuously updated product characteristics and manufacturing technologies.

Adaptive process design models can adjust process parameters in real time, ensuring that product performance meets predefined standards despite fluctuations in raw material supply, changes in environmental conditions, and variations in production equipment performance. These models, through real-time data collection, advanced the data processing technology, and machine learning algorithms can monitor and predict the impact of key process parameters on product performance, making immediate adjustments when deviations are detected. In a production mode of multiple varieties and small batches, they can quickly switch between different products, reducing the time and cost to set new process parameters, thereby increasing production flexibility and efficiency. As such, adaptive process design models can learn and predict the process conditions required for new product designs, quickly adapt to changes on the production line, reduce downtime, and enhance the overall production capacity.

With the advancement of manufacturing technology, especially in Industry 4.0, the importance of adaptive process design models will be further enhanced to support more intelligent and automated production systems. Thus, the demand for adaptability in product quality prediction models is also growing. The core component of the adaptive process design model is the adaptability of the product quality prediction model. Transfer learning is an important pathway to improve the adaptability of product prediction models. In this work, we focus on representation-level adaptability, which motivates the use of feature-encoded transfer learning. This strategy allows invariant process representations to be learned from historical data while preserving flexibility for adaptations under new working conditions with minimal additional data.

## 4. Transfer Learning-Based Method

**Figure 3:**
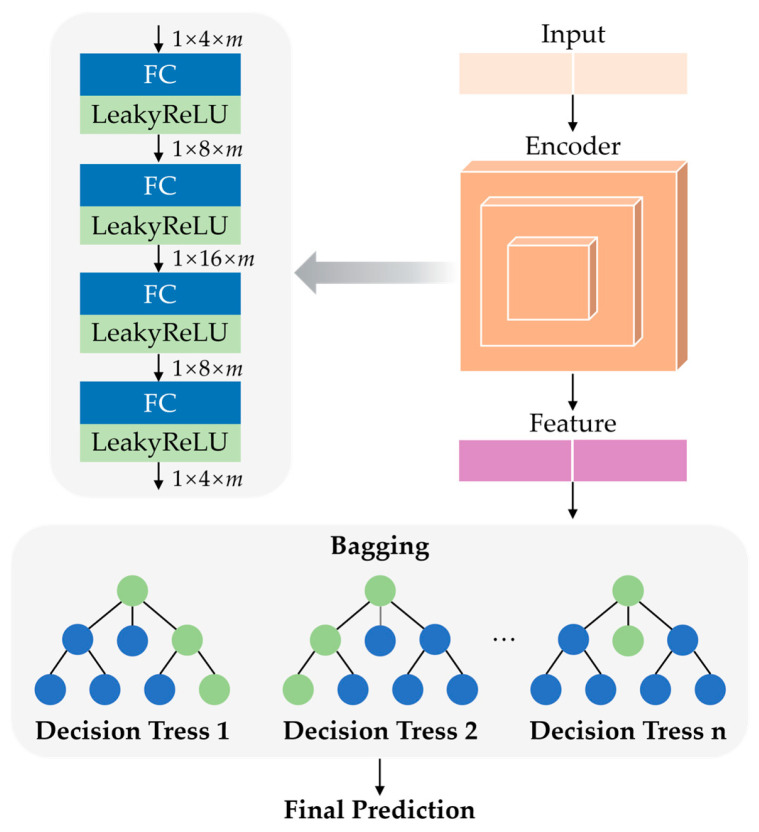
The architecture of proposed network.

This section introduces a feature encoding-based transfer learning prediction model for predicting surface roughness, which takes into account the workpiece material (i.e., 316L stainless steel 3D printed parts), polishing parameters (feed rate (f), fluid pressure (P), tool offset (TO), and distance (d)), as well as surface roughness parameters (i.e., initial surface roughness, *Ra_i_*, and final surface roughness, *Ra_f_*). Figure 3 illustrates the framework of the proposed feature-encoded transfer learning (FETL) model. The model consists of two core modules: (a) the feature encoding module and (b) the regression prediction module. After encoding the input data in the feature encoding module, the generated feature values are passed to the regression prediction module, where the final predicted surface roughness is determined through the guidance aggregation of multiple sub-decision trees. Therefore, domain-invariant representations can be extracted to preserve essential process–quality relationships while suppressing task-specific variations to avoid performance degradation due to distribution shifts caused by changes in materials, nozzle configurations, and process parameters.

### 4.1. Transfer Learning Strategy

During the process of polishing different workpiece materials, there is a shift in the data concerning polishing parameters and surface roughness. When the same model is used to perform both tasks simultaneously, this shift can lead to a significant reduction in model accuracy. To make full use of polishing data from different materials and processes, a feature encoding-based transfer learning strategy is proposed in this paper. This strategy enables the model to transfer from the labeled data of different tasks (source domain data) to the unlabeled data of the new task (target domain data), thus training a model suitable for the target domain. The core idea of this strategy is that an encoder is designed to process the input data and learn a domain-invariant feature representation. The encoder can be intuitively understood as a process-aware transformation that maps heterogeneous polishing conditions into a unified latent space, enabling the regression model to focus on the fundamental polishing mechanism. In addition, the effectiveness of the proposed transfer strategy depends on the similarity of the underlying process physics between the source and target domains.

**Figure 4:**
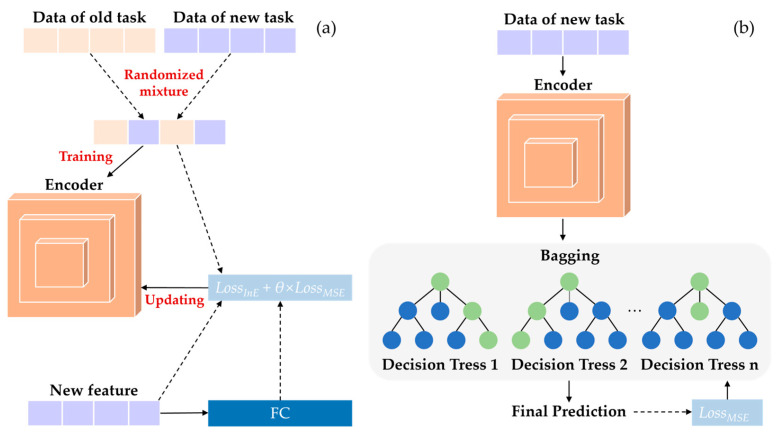
The training process of the prediction model in (**a**) stage 1: feature encoder training using mixed source and target domain data to learn shared representations; and (**b**) stage 2: regression model training based on encoded features for surface roughness prediction.

As shown in Figure 4, the training process of the prediction model is divided into two stages. In the first stage, the data from the source and target domains are randomly mixed and then input into the feature encoding module to learn a common representation of the data between domains. In the second stage, using the encoding module trained in the first stage, the feature representation of the current task’s input data is obtained and passed into the regression prediction module, which learns the mapping between the encoded features and the predicted values. When training the encoding module, the loss function is designed to focus on the information loss between the encoded feature and the original input, guiding the learning direction of the encoding module and eliminating the impact of model overfitting on the target domain’s feature recognition ability.

The training of the encoder module consists of forward propagation and backward parameter tuning processes. Assuming that the network processes m training samples ${\left\{\left(x^{\left(i\right)},y^{\left(i\right)}\right)\right\}}_{i=1}^{m}$ in one batch, where $x^{\left(i\right)}\epsilon R^{4}$ is the input vector and $y^{\left(i\right)}\;$is the labeled final surface roughness for that sample, then the forward propagation calculates the input features at each layer as follows:

(1) $$x^{l}=f\left(W^{l}x^{l-1}+b^{l}\right)$$

where $x^{l-1}$ and $x^{l}\;$denote the input and output feature vectors of the l-th layer, respectively; $W^{l}$ and $b^{l}\;$are the weights and bias vectors of the neurons in the *l*-th layer network, respectively. f(·) is the activation function defined as follows:

(2) $$LeakyReLU\left(x\right)=\left\{\begin{array}{c} x,x\geq 0\\ \eta x,x<0 \end{array}\;\right.$$

where ηϵ(0,1) is a predefined constant. For the training set containing m samples, the overall objective function of the encoder module is defined as follows:

(3) $$J(W,b)=\frac{1}{m}\sum_{i=1}^{m}\frac{1}{2}{\left\|h_{W,b}{(x}^{\left(i\right)})-y^{\left(i\right)}\right\|}^{2}+\frac{\lambda }{2}\sum_{l=1}^{n_{l}-1}\sum_{i=1}^{s_{l}}\sum_{j=1}^{s_{l}+1}{\left(W_{ij}^{\left(l\right)}\right)}^{2}\;$$

Here, *λ* stands for the weight attenuation parameter, $n_{l}$ is the total number of layers in the network, $s_{l}$ is the total number of neurons in the *l*-th layer of the network, $h_{W,b}{(x}^{\left(i\right)})$ is the total output of the encoding module under the current weight W and bias b, and $W_{ji}^{\left(l\right)}$ is the weight of neurons in the *l*-th layer.

During backpropagation, the parameters are updated using gradient descent:

(4) $$W_{ij}^{\left(l\right)}=W_{ij}^{\left(l\right)}-\alpha \frac{\partial }{\partial W_{ij}^{\left(l\right)}}J\left(W,b\right)$$

(5) $$b_{i}^{\left(l\right)}=b_{i}^{\left(l\right)}-\alpha \frac{\partial }{\partial b_{i}^{\left(l\right)}}J\left(W,b\right)$$

where α is the learning speed.

When the cost error of the entire network structure is minimized, a pre-trained encoder module is obtained. The encoder module proposed in this paper consists of four basic blocks connected in series, each block containing a fully connected layer and an activation function layer. The input data are 1 × 4 × *m* vectors, and the first two blocks gradually expand to 1 × 8 × *m* and 1 × 16 × *m* vectors, respectively. Then, through the last two blocks, the vector is gradually compressed back to 1 × 8 × *m* and finally to a 1 × 4 × *m* feature vector. In the end, the encoding module outputs a feature vector whose dimensions are the same as the original input data. The encoder is optimized jointly with the surface roughness prediction objective and a cross-domain information constraint to learn task-relevant and domain-invariant feature representations.

The training of the regression prediction module consists of four parts: data preparation, decision tree construction, ensemble prediction, and parameter updating. First, the original input data are processed using the trained encoding module from stage one, which provides the input data for the regression prediction module. Second, a random training sample set is obtained using a bootstrapping sampling method, and fully split child decision trees are built. Then, the final predicted surface roughness is determined through the ensemble of multiple sub-decision trees. Finally, the predictions are compared with the actual values, and the parameters of the regression prediction module are updated accordingly.

### 4.2. Loss Function

**Loss function for stage 1**: The purpose of the first stage of training is to enable the encoding module to learn a feature expression that is invariant across domains from the mixed data of the target and source domains, in order to better accomplish the surface roughness prediction task. However, for this feature expression, it is not possible to obtain true label values to calculate the loss function. Therefore, this paper focuses on the error of the final prediction task, as well as the information loss between the feature and the original input data, designing the following loss function:

(6) $${Loss}_{1}={Loss}_{InE}+\theta {Loss}_{MSE}$$

where ${Loss}_{InE}$ is information loss, ${Loss}_{MSE}$ is the mean squared error loss, and θ is the balancing weight coefficient. The information loss is used to measure how much information is lost when the feature expression is used; expressing the similarity between the two is implemented using the Kullback–Leibler (KL) divergence as shown in Equation (7):

(7) $${Loss}_{InE}=\sum_{i=1}^{d}p_{i}ln\frac{p_{i}}{q_{i}}=D_{KL}\left(p∥q\right)$$

where d is the feature dimension and $p_{i}$ and $q_{i}$ represent the probabilities associated with the i-th feature component in the target and source domains, respectively. The corresponding probability distributions $p=\{p_{i}\}$ and $q=\{q_{i}\}$ are obtained by applying Softmax normalization to the feature vectors as shown in Equation (8):

(8) $$p_{i}=\frac{\exp\left(f_{i}\right)}{\sum_{j=1}^{d}\exp\left(f_{j}\right)},\;q_{i}=\frac{\exp\left(g_{i}\right)}{\sum_{j=1}^{d}\exp\left(g_{j}\right)}$$

where $f=\left\{f_{i}\right\}$ and $g=\{g_{i}\}$ denote the encoded feature vectors extracted from the target and source domains. The mean squared error (MSE) loss calculates the square difference between the predicted values and the target values, defined in Equation (9):

(9) $${Loss}_{MSE}=\frac{1}{n}\sum_{i=1}^{n}{\left(y_{i}-{\hat{y}}_{i}\right)}^{2}\;$$

In the first stage of training, the input to the encoding module is passed to the fully connected layer, and its output is used as the final predicted surface roughness, which is then used to calculate the MSE loss with ground-truth. This is weighed up with the information loss calculated from the encoded features to obtain the overall total loss. The model parameters of each block of the encoding module are updated through backpropagation.

**Loss function for stage 2**: The purpose of the second stage of training is to enable the regression prediction module to learn the mapping between the encoded features and the predicted surface roughness. During the model training process, it is only necessary to focus on the error of the prediction task, and therefore the MSE loss function is used as the overall loss function for stage two.

## 5. Experimental Verification

### 5.1. Experimental Setup

**Figure 5:**
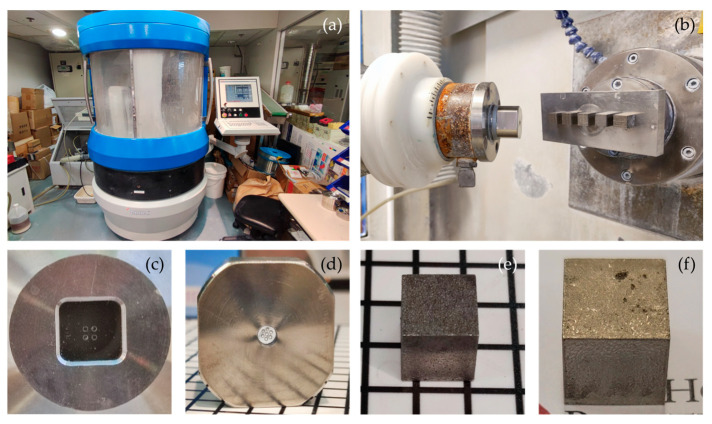
Experiment setup for multi-jet polishing. (**a**) The ZEEKO IRP 200 polishing equipment, (**b**) the polishing setting, (**c**) the 4-jet nozzle, (**d**) the 7-jet nozzle, (**e**) the 316L stainless steel workpiece, and (**f**) the CoCr workpiece.

During the MJP experiment, the ZEEKO IRP200 machine (Zeeko Limited, Coalville, UK) was used for polishing with different process parameters, as shown in Figure 5. Two different working conditions were setup, using seven nozzles and four nozzles, respectively (see Figure 5c,d). The polishing setup is shown in Figure 5b, with 3D printed 316L stainless steel components and CoCr (see Figure 5e,f). In addition, the explicit process parameters used by the predictive model are listed in Table 1. Process factors such as nozzle configuration and jet kinetic energy are treated as domain-level conditions, and their effects are incorporated through transfer learning across different domains. After polishing, the surface roughness (*Ra*) was measured three times and averaged to obtain the final Ra. For the two conditions, 43 and 80 sets of experimental datasets with different polishing parameters were collected, including feed rate (*f*), fluid pressure (*P*), tool offset (TO), step size (*d*), initial surface roughness (*Ra_i_*), and surface direction. All experimental data were collected under the assumption that the sensing environment remained stable. The process parameters are treated as continuous or quasi-continuous variables, and the predictive model aims to learn an underlying process–quality mapping from the sparsely sampled data. For the target domain, the dataset is first split into training and testing subsets. During Stage 1, the encoder is trained using a mixed dataset consisting of the source domain data and the training subset of the target domain only. The target domain test data are not used in any stage of encoder training. During stage 2, the regression model is trained using the encoded features from the target domain training set and evaluated exclusively on the held-out target domain test set. The dataset covers different materials, nozzle configurations, and initial surface states, which provides a basis for evaluating cross-condition adaptability.

Our model was implemented in PyTorch and optimized using the Adam optimizer, with the parameters set to *β*_1 = 0.9 and *β*_2 = 0.999. The proposed model was compiled on a computer server equipped with a Tesla V100 PCIe 32 GB GPU card (NVIDIA Corporation, Santa Clara, CA, USA), using Python 3.6, Pytorch 1.8, and CUDA 10.2.

### 5.2. Surface Topography Analysis Before and After Multi-Jet Polishing

**Figure 6:**
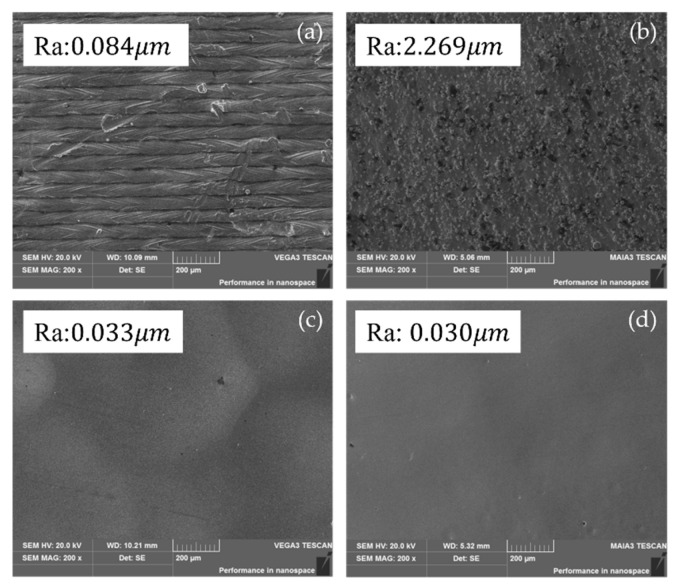
Scanning electron microscope images of the 316L workpiece before and after multi-jet polishing. (**a**) The top surface (Ra: 0.084 μm) and (**b**) side surface (Ra: 2.269 μm) of workpiece before polishing, respectively. (**c**) The top surface (Ra: 0.033 μm) and (**d**) side surface (Ra: 0.030 μm) of workpiece after polishing, respectively.

The surface quality of 316L stainless steel and CoCr after the 3D printing process were evaluated. As shown in Figure 6, a clear observation from the before-and-after comparison indicates that the laser melting traces produced during the 3D printing process were effectively eliminated by the MJP procedure. Moreover, surface defects such as un-melted particles and cracks were also significantly improved. However, based on the surface comparison scanning electron microscope (SEM) results before and after polishing, there are still some larger-scale wave-like textures present. This is because the micrometer-sized particles in the abrasive media used during polishing can only remove roughness to a certain extent and are not effective for millimeter- or sub-millimeter-level irregularities.

### 5.3. Comparison with State-of-the-Art Methods

**Figure 7:**
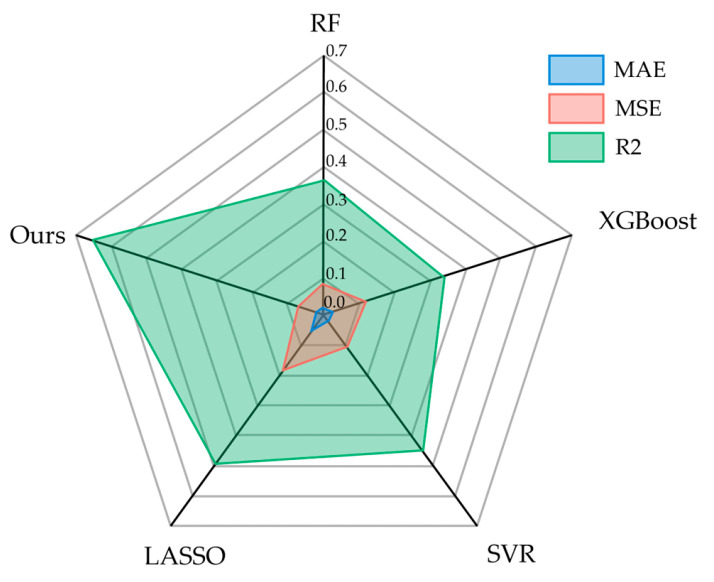
Comparison of prediction accuracy with SOTA model.

Comparison experiments were conducted with state-of-the-art (SOTA) methods, including XGBoost, RF (Random Forest), LASSO (Least Absolute Shrinkage and Selection Operator), and SVR (Support Vector Regression). The numerical results of these methods are presented in Figure 7. Our method outperformed in all three metrics: MAE (Mean Absolute Error), MSE, and R2 (Coefficient of Determination). MAE, MSE, and R2 are metrics used to measure the predictive capability of regression models. They quantify the model’s prediction accuracy by calculating the differences between the true values and the predicted values.

MAE is less sensitive to outliers, but it does not reflect the distribution of predictive errors. MSE is sensitive to outliers (because when the error is greater than 1, squaring it will further amplify the value), but it can reflect the distribution of predictive errors. R2 indicates the proportion of the variance for the dependent variable that is explained by the model; its value ranges from 0 to 1, with values closer to 1 indicating a better fit of the model to the data.

As shown in Figure 7, our method achieved the lowest MAE and MSE, indicating the smallest discrepancy with the true values. The highest MAE and MSE were observed for LASSO, indicating the lowest prediction accuracy. The other three methods had similar metric values, suggesting a comparable level of discrepancy between their predictions and the actual values. From the R2 metric perspective, our model had the best fit to the data, while XGBoost had the poorest fit. The results also indicate that the prediction errors calculated by MAE and MSE do not fully represent the model’s fitting ability.

### 5.4. Ablation Experiments

In this section, an ablation study will be conducted to determine the effectiveness of the main innovations in the proposed method. Specifically, the impact of the loss function, transfer strategy, and feature transformation [30] on performance improvement was studied. The quantitative results of the ablation experiments are shown in Table 2. By learning shared feature representations, the model can effectively reuse information across materials and nozzle configurations, alleviating performance degradation caused by limited target domain samples.

**Figure 8:**
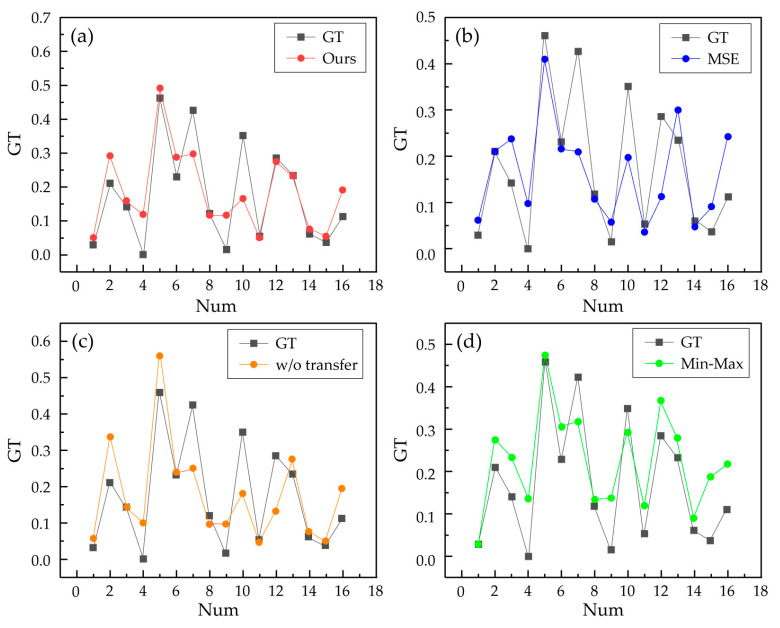
Results of ablation test. (**a**) Ours, (**b**) MSE loss, (**c**) w/o transfer, and (**d**) min–max.

Compared to using only MSE loss, our method reduced MAE (Mean Absolute Error) by 0.01 and increased R2 by 0.021, with no change in MSE. Training with MSE loss directly targets the reduction in mean squared error, which is sensitive to outliers, so the model training results tend to eliminate outliers, which may lead to a homogenization of prediction results and not learning the true distribution of the data. Compared to not using a transfer strategy, R2 increased by 0.19, MAE decreased by 0.015, and MSE decreased by 0.04. The purpose of the transfer is to make full use of the existing data, applying the knowledge learned from similar data to new tasks, which is why there is a significant improvement in all three evaluation metrics. Compared to the common min–max feature transformation method, the proposed method has improvements in both MAE and R2, but the extent of improvement is smaller compared to others. Figure 8 shows the fit of different models to each sample in the ablation study, where Figure 8a reports point predictions obtained by averaging the outputs of the bagging ensemble of our proposed model.

## 6. Conclusions

In conclusion, this study presents a digital twin (DT)-driven process design framework for multi-jet polishing (MJP), aiming to address the challenges associated with complexity and uncertainty under changing working conditions. The proposed framework focuses on enhancing decision support during process design through improved quality prediction.

The human-centric design approach integrates critical factors of DT-driven MJP, including jet kinetic energy, nozzle structure, abrasive type, and machining path. In particular, the incorporation of a feature-encoded transfer learning strategy enables more accurate and robust surface roughness prediction across different polishing conditions by embedding standard encoding architectures into precision manufacturing workflows. The framework provides a generalizable approach for improving robustness and efficiency in data-scarce and uncertainty-dominated machining environments. The validation experiments on 3D printed workpieces were conducted to demonstrate the effectiveness.

Although the proposed transfer learning-based method demonstrates strong predictive performance, its applicability under increasing source–target discrepancies has not been systematically explored. In future work, we will evaluate the generality of the proposed framework in other ultra-precision manufacturing processes, such as bonnet polishing, chemical mechanical polishing, and magnetic abrasive finishing, as well as the integration of real-time system deployment [7], human–machine interaction interfaces, and trustworthy physical–virtual consistency under evolving sensing configurations.

## Figures and Tables

**Table 1:** Explicit process parameters used as model inputs under different nozzle configuration domains.

Parameter	Working Condition 1	Working Condition 2
Injector	7	4
Material	316L stainless steel	CoCr
Feed rate size (mm/min)	10, 15, 20, 25, 30, 40, 60, 80	10, 15, 20, 25, 30, 40, 60, 80
Fluid pressure (bar)	4, 5, 6, 7, 8, 9, 10	4, 5, 6, 7, 8, 9, 10
Tool offset (mm)	2.5, 5, 7.5, 10, 12.5, 15	2.5, 5, 7.5, 10, 12.5, 15
Step size (mm)	0.1, 0.2, 0.3, 0.4, 0.5, 0.6, 0.7, 0.8	0.1, 0.2, 0.3, 0.4, 0.5, 0.6, 0.7, 0.8
Initial surface roughness (nm)	400~500	2000~4200
Surface direction	TS (Top)	TS (Top)

**Table 2:** Ablation test.

Methods	MAE	MSE	R^2^
Ours	0.066	0.007	0.643
Min–Max	0.073	0.007	0.616
w/o Transfer	0.081	0.011	0.453
MSE Loss	0.076	0.007	0.622

## Data Availability

The original contributions presented in this study are included in the article. Further inquiries can be directed at the corresponding author.

## Abbreviations

The following abbreviations are used in this manuscript:

DT	Digital Twin
RF	Random Forest
SVR	Support Vector Regression
MJP	Multi-Jet Polishing
MAE	Mean Absolute Error
FJP	Fluid Jet Polishing
UPM	Ultra-Precision Machining
LASSO	Least Absolute Shrinkage and Selection Operator
R2	Coefficient of Determination
MSE	Mean Square Error
